# Policies and priorities to combat NCD challenges in India

**DOI:** 10.1186/1472-6963-14-S2-P114

**Published:** 2014-07-07

**Authors:** Ruchi Sogarwal, Damodar Bachani

**Affiliations:** 1 MAMTA Health Institute for Mother and Child, New Delhi, Delhi, India; 2 Lady Hardinge Medical College, New Delhi, Delhi, India

## 

Chronic non-communicable diseases (NCDs) have replaced communicable diseases as the most common causes of morbidity and premature mortality worldwide. Since 1980, the Government of India had supported states in prevention and control of cancer as a vertical program. However, several challenges were identified, such as: lack of comprehensive approach to key NCDs including diabetes and cardiovascular diseases; limited emphasis on health promotion and preventive measures to reduce exposure to risk factors; lack of facilities and capacity for screening, early diagnosis and effective management within the public health care system was not adequately addressed. Aiming at preventing rise of NCDs, the government launched the National Programme for Prevention and Control of Cancer, Diabetes, Cardiovascular Diseases and Stroke (NPCDCS) in 2010 and planned to expand to all districts of the country in a phased manner. The current programme has prioritised preventive and promotive services to the general population and holistic care to the people with NCDs at primary, secondary and tertiary levels of health-care with integrated management and strong monitoring system for making services universally accessible in the country. Under the mass screening initiative, nearly 60 million people aged 30 years and above were screened for diabetes and hypertension. Those identified with raised blood sugar and high blood pressure need to be referred for further investigations, treatment and periodic follow-up. It is therefore recommended that the revised National Health Policy should duly emphasize policies and strategies to prevent and control key NCDs through population-based interventions that require multi-sectoral approach. The referral system needs strengthening and secondary and tertiary levels of health care require further strengthening key NCDs and their complications. Due attention is to be given for implementation of these programs in remote rural areas and underprivileged population in the urban areas. For India, having committed to global indicators and goals to address NCDs and risk factors by the year 2025, a missionary approach is required for effective implementation of programs.

